# Uncommon radiologic computed tomography appearances of the chest in patients with lymphangioleiomyomatosis

**DOI:** 10.1038/s41598-021-85999-5

**Published:** 2021-03-30

**Authors:** Yasuhito Sekimoto, Kazuhiro Suzuki, Makiko Okura, Takuo Hayashi, Hiroki Ebana, Toshio Kumasaka, Keiko Mitani, Koichi Nishino, Shouichi Okamoto, Etsuko Kobayashi, Kazuhisa Takahashi, Kuniaki Seyama

**Affiliations:** 1 Division of Respiratory Medicine, Juntendo University Faculty of Medicine and Graduate School of Medicine, 3-1-3 Hongo, Bunkyo-ku, Tokyo, 113-8431 Japan; 2 Division of Radiology, Juntendo University Faculty of Medicine and Graduate School of Medicine, 3-1-3 Hongo, Bunkyo-ku, Tokyo, 113-8431 Japan; 3 Division of Human Pathology, Juntendo University Faculty of Medicine and Graduate School of Medicine, 2-1-1 Hongo, Bunkyo-ku, Tokyo, 113-8421 Japan; 4 The Study Group for Pneumothorax and Cystic Lung Diseases, 4-8-1 Seta, Setagaya-Ku, Tokyo, 158-0095 Japan; 5 Department of Respiratory and Cardiovascular Surgery, Tokyo Metropolitan Bokuto Hospital, 4-23-15 Kotobashi, Sumida-ku, Tokyo, 130-8575 Japan; 6 Department of Pathology, Japanese Red Cross Medical Center, 4-1-22 Hiroo, Shibuyaku, Tokyo 150-8935 Japan

**Keywords:** Respiratory tract diseases, Respiratory signs and symptoms, Diagnosis

## Abstract

Lymphangioleiomyomatosis (LAM) is a rare destructive lung disease characterized by multiple thin-walled pulmonary cysts. The currently proposed diagnostic algorithm emphasizes the characteristic cystic appearance on high-resolution computed tomography (HRCT) so uncommon HRCT appearances present challenges to establishing the proper LAM diagnosis. The objective of this study is to accrue uncommon chest HRCT appearances, determine frequencies in both tuberous sclerosis complex (TSC)-associated LAM (TSC-LAM) and sporadic LAM (S-LAM) patients. 311 females referred to our hospital, including 272 S-LAM patients (mean age 39.2 years) and 39 TSC-LAM patients (mean age 38.3 years), were retrospectively evaluated. We found 2 types of radiologic findings likely to make HRCT cyst appearance atypical: characteristics of the cyst itself and uncommon findings in addition to cysts. We found that approximately 80% of LAM patients, whether TSC-associated or sporadic, showed typical HRCT appearance with mild to severe cystic destruction. The remaining 20% displayed unusual profiles in cyst appearance as well as additional findings aside from cyst: the former includes large cyst, thickened walls, and irregularly shaped whereas the latter includes ground glass attenuation and diffuse noncalcified nodules. It is important to be aware of various radiologic findings that make HRCT cystic appearance atypical of LAM.

## Introduction

Lymphangioleiomyomatosis (LAM) is a rare cystic lung disease that typically affects women of childbearing age and presents as either tuberous sclerosis complex (TSC)-associated LAM (TSC-LAM) or sporadic LAM (S-LAM)^1^. LAM is a progressive disease which eventually leads to respiratory failure. However, the Multicenter International Lymphangioleiomyomatosis Efficacy and Safety of Sirolimus (MILES) trial revealed that sirolimus, a mechanistic target of rapamycin inhibitor, stabilizes lung function, reduces serum vascular endothelial growth factor-D (VEGF-D) levels, and ameliorates symptoms^2^. Therefore, the diagnosis of LAM should be precisely established prior to the initiation of sirolimus therapy.

In this context, an official American Thoracic Society (ATS)/Japanese Respiratory Society (JRS) Clinical Practice Guideline has been established and presents a standard algorithm to make a diagnosis of LAM^3^. In the diagnostic workflow, the evaluation of high-resolution computed tomography (HRCT) of the chest is indicated as a second step after the clinical suspicion of LAM, to examine whether the pathognomonic cystic appearance, i.e., multiple thin-walled oval-shaped pulmonary cysts distributed evenly throughout both lung fields, is found. Thus, HRCT appearance becomes the gateway for pulmonologists to establish the diagnosis of LAM^3^. However, the diagnostic accuracy is reported to be approximately 90% by expert radiologists and pulmonologists^4^. Although this figure appears to be sufficiently high, about 10% of patients might be missed. We speculate that this is caused by the fact that some patients with LAM may not show the common cystic appearance on HRCT at presentation and therefore may pose diagnostic challenges. The aim of this study is to compile a list of uncommon HRCT findings that make it difficult to recognize the pathognomonic HRCT appearance of LAM, determine their frequencies, and investigate differences in frequency of these uncommon findings between TSC-LAM and S-LAM patients where applicable.

## Methods

We retrospectively evaluated radiologic findings of chest HRCT (with a section thickness of 2 mm) from 311 females, including 272 patients with S-LAM (mean age 39.2 years [range 19–71]) and 39 patients with TSC-LAM (mean age 38.3 years [range 21–66]) who were seen at our hospital between April 2009 and December 2016 (Table 1). The diagnosis of LAM was established by the diagnostic algorithm proposed by the ATS/JRS Clinical Practice Guideline^3^: either histopathologic findings or the combination of distinct clinical manifestations and/or serum VEGF-D > 800 pg/ml. The diagnosis of TSC was established according to the international consensus guideline^5^. All chest HRCT scans were obtained at the end of inspiration by patients who were in a supine position. No intravenous contrast material was used. Histopathological examinations and immunohistochemistry of the lungs were performed as previously reported^6^.

**Table 1:** Frequencies of chest HRCT findings in study participants.

	Total (n = 311)	S-LAM (n = 272) n (%)	TSC-LAM (n = 39) n (%)	*P* value
**Age at presentation** (y) Mean ± SD	39.1 ± 9.4	39.2 ± 9.2	38.3 ± 10.3	*P* = 0.481
Median (range)	38 (19 – 71)	38 (19 – 71)	36 (21 – 66)	
**Cyst appearance**				
Common appearance* alone	254 (81.7%)	223 (82.0%)	31 (79.5%)	*P* = 0.706
Common appearance + large cysts†	52 (16.7%)	44 (16.2%)	8 (20.5%)	*P* = 0.497
Common appearance + cysts with irregularly thickened walls	2 (0.6%)	2 (0.7%)	0	
Multiple thin-walled cysts, mostly large	2 (0.6%)	2 (0.7%)	0	
Multiple thin-walled cysts, mostly irregularly shaped	1 (0.3%)	1 (0.4%)	0	
**Findings in addition to cysts**				
Lymphatic congestion	24 (7.7%)	20 (7.4%)	4 (10.3%)	
Lobar area	16 (5.1%)	12 (4.4%)	4 (10.3%)	
Limited area	6 (1.9%)	6 (2.2%)	0	
Mediastinal area	2 (0.6%)	2 (0.7%)	0	
Diffuse noncalcified nodules^¶^	6 (1.9%)	6 (2.2%)	0	
with small cavitary changes	4 (1.3%)	4 (1.5%)	0	
with ground-glass attenuation	3 (1.0%)	3 (1.1%)	0	
TSC-related findings				
Rounded ground-glass opacities suggestive of MMPH	15 (4.8%)	1 (0.4%)	14 (35.9%)	*P* < 0.001
Myocardial fatty foci	26 (8.4%)	8 (2.9%)	18 (46.1%)	*P* < 0.001
Bone nodules	60 (19.3%)	28 (10.3%)	32 (82.1%)	*P* < 0.001

*HRCT* high resolution computed tomography, *LAM* lymphangioleiomyomatosis, *MMPH* multifocal micronodular pneumocyte hyperplasia, *SD* standard deviation, *S-LAM* sporadic LAM, *TSC-LAM* tuberous sclerosis complex-associated LAM, *y* years of age.

*Common appearance is multiple thin-walled oval-shaped pulmonary cysts distributed evenly throughout both lung fields.

^†^Large cysts are those > 2 cm.

^¶^One patient had diffuse nodules with both small cavitary changes and ground-glass opacities.

### The classification of HRCT findings of the chest

We classified radiologic findings into 2 categories: cyst appearance and findings in addition to cysts. We found 3 types of uncommon cyst appearances: large cyst which was arbitrarily defined as a cyst > 2 cm in diameter, cyst with irregularly thickened walls, and cyst with irregularly shaped. On the other hand, we found 2 types of findings in addition to cysts: ground glass attenuation suggestive of lymphatic congestion and diffuse noncalcified nodules. We also evaluated the frequency of TSC-related findings in S-LAM. TSC-related findings include rounded ground-glass opacities suggestive of multifocal micronodular pneumocyte hyperplasia (MMPH), myocardial fatty foci (MFF) and bone nodules.

### Statistical analysis

Non-parametric variables were compared between S-LAM and TSC-LAM patients using the Mann–Whitney U test. Comparisons between all patients and various subgroups were conducted using the Fisher’s exact tests. All statistical analyses were performed using the SPSS software program (version 25.0 SPSS Inc., Chicago, IL, USA). A *P*-value of less than 0.05 was considered significantly different.

### Ethics approval and consent to participate

This study was approved by the Institutional Review Board (IRB) of the Juntendo Hospital (IRB No. 19-133) and performed in accordance with the Declaration of Helsinki and the relevant guidelines and regulations. The Ethical Committee waived the requirement for informed consent because of the retrospective nature of the study.

## Results

### Chest HRCT findings in study participants

Age and HRCT findings of the chest are presented in Table 1. There was no significant difference in age at presentation between S-LAM and TSC-LAM patients. Table 1 shows frequencies of the key subgroups found within each of these 2 dimensions which are described below. Representative HRCT images and corresponding histopathologic photomicrographs are also presented when they were available. Clinical features including type of diagnosis (histopathological or clinical), age at presentation, and occurrence of pneumothorax stratified by image finding are shown in Table S1.

### Cyst appearance

#### Common cystic appearance

**Figure 1:**
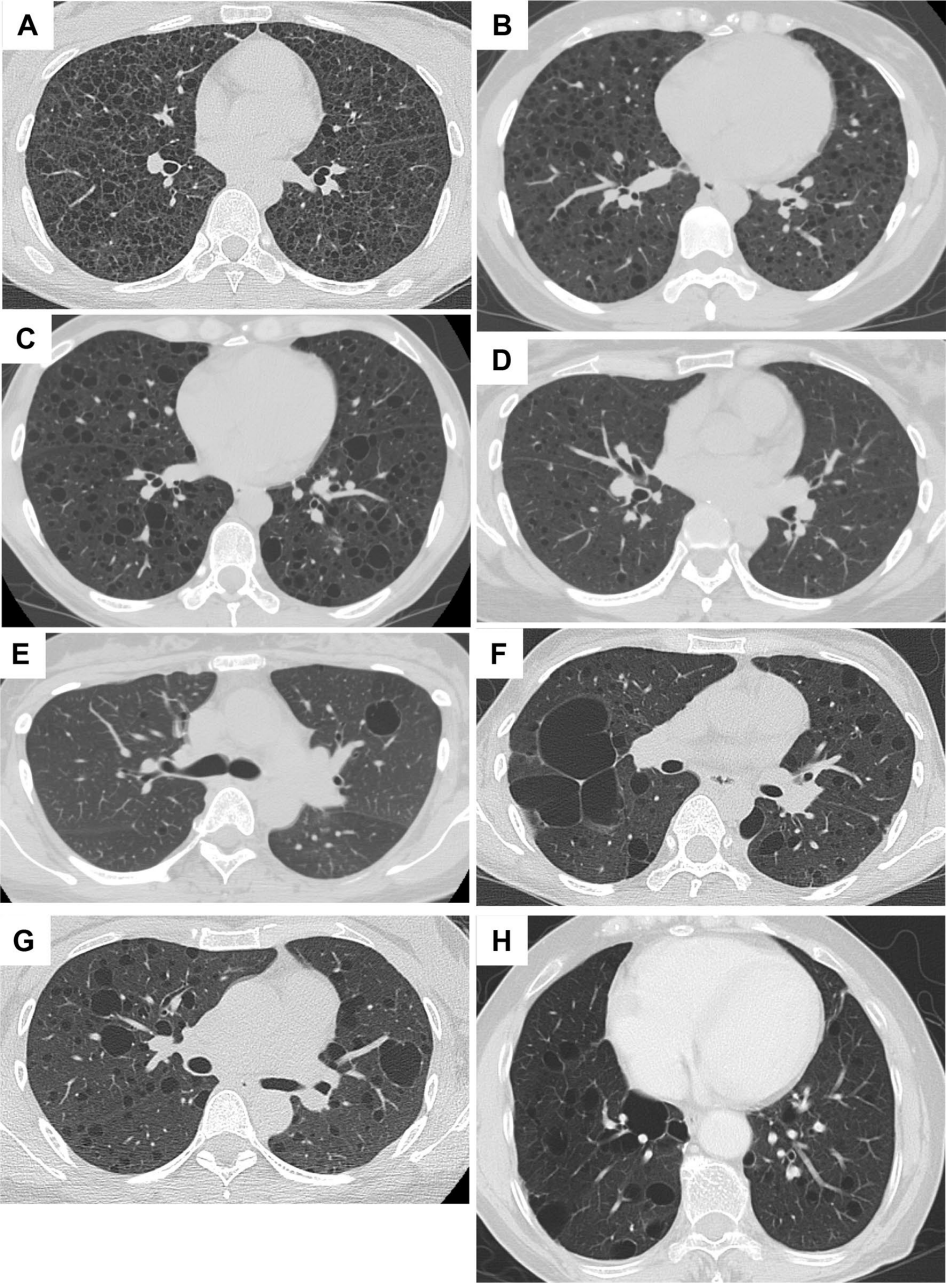
Representative HRCT images of common cystic appearance alone or with co-existence of large cysts. (**A**) A 32-year-old female with S-LAM whose diagnosis was established by transbronchial lung biopsy (TBLB). Numerous pulmonary cysts are noted. (**B**) A 33-year-old female, clinically diagnosed as having S-LAM with moderate to severe cyst formation. (**C**) A 45-year-old female with S-LAM, diagnosed by lung biopsy, showed mild cyst formation. (**D**) A 39-year-old female with S-LAM, diagnosed by lung biopsy, showed very mild cyst formation. (**E**) A 39-year-old female with S-LAM. Cyst formation was very mild but included a large cyst. She had a history of right pneumothorax and LAM was diagnosed by lung biopsy. (**F**) A 38-year-old female with S-LAM. Large irregularly shaped cysts were observed in the right lung along with the pathognomonic cystic appearance. She had bilateral pneumothoraces simultaneously and the diagnosis was made histopathologically. (**G**) A 41-year-old female with S-LAM. Abnormality on chest roentgenogram was discovered at a regular health check-up and the diagnosis was made by lung biopsy. Several large cysts were seen in both lung fields. (**H**) A 71-year-old female with S-LAM. She had a right lower lobectomy due to lung cancer, but the co-existence of LAM in underlying lung tissue was not recognized. Large cysts were observed more frequently in the remaining right lung than the left lung.

The common cystic appearance of LAM is multiple well-circumscribed, thin-walled, oval-shaped cysts, usually less than 2 cm in size, distributed evenly throughout both lung fields. This was found in 223 (82.0%) of the 272 patients with S-LAM and 31 (79.5%) of the 39 patients with TSC-LAM (Table 1). The representative HRCT images from S-LAM patients with cyst formation ranging from mild to severe are shown in Fig. 1A–D.

#### Common appearance with co-existence of large cysts > 2 cm

Some LAM patients had uncommonly large cysts in addition to the common cystic appearance on HRCT images. This manifestation was found in 44 (16.2%) of the S-LAM patients and 8 (20.5%) of the TSC-LAM patients (Table 1). Representative HRCT images from 4 S-LAM patients are shown in Fig. 1E–H. The large cysts were irregularly shaped or distorted (Fig. 1F) and often distributed to the basal or mediastinal side, or the subpleural area of lung fields (Figs. 1F,H, 4A,B). We found that LAM patients who had the common cystic appearance with co-existence of large cysts included a significantly larger proportion of patients who had developed pneumothorax before or at the diagnosis of LAM compared to the all patients (80.8% vs. 38.3%, *P* < 0.0001) (Table S1). This subgroup also tended to have a higher proportion of LAM patients ≥ 50 years of age at onset (or their initial visit) than all study participants combined, although it’s not statistically significant (23.1% vs. 14.5%, *P* = 0.0809) (Table S1).

#### Common appearance with co-existence of cysts with irregularly thickened walls

**Figure 2:**
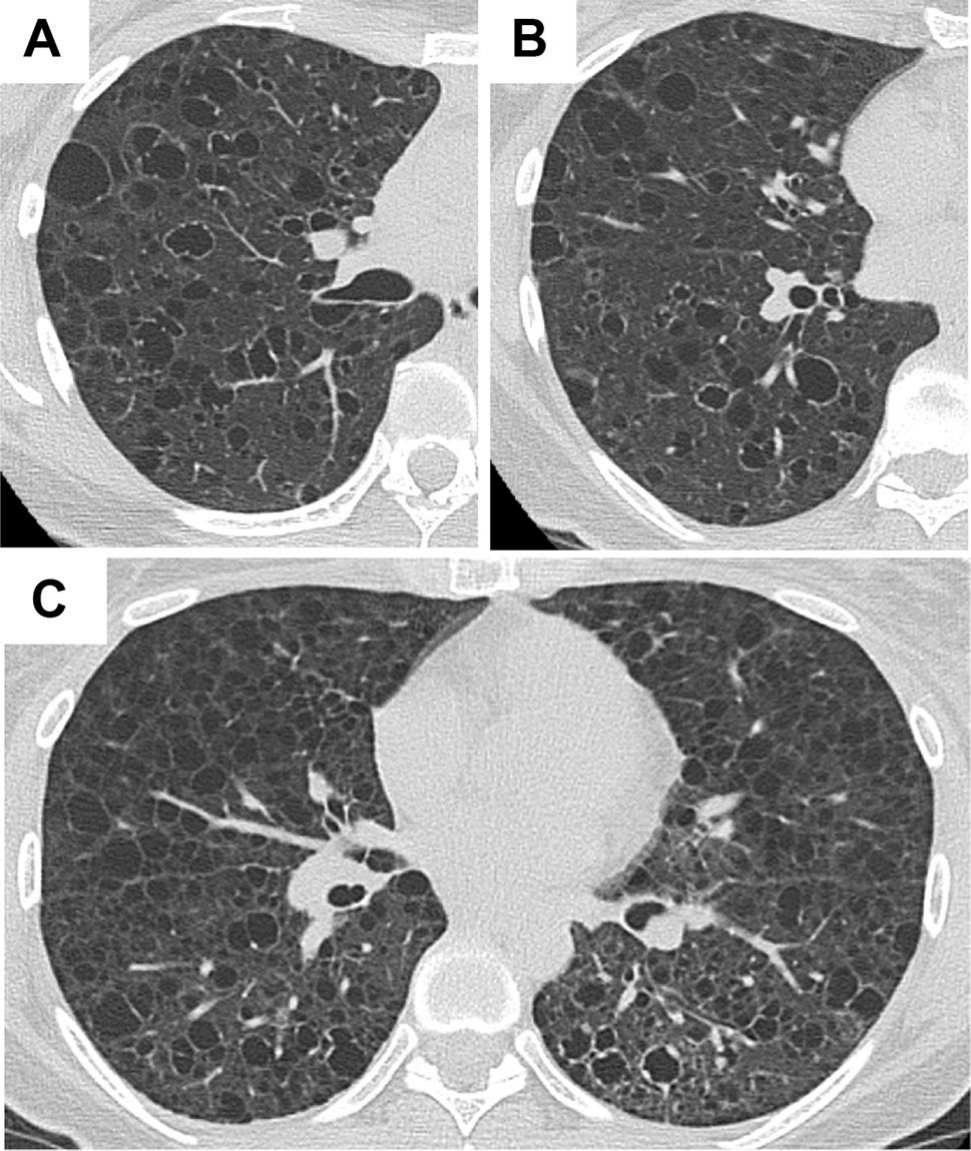
Representative HRCT images with common appearance and co-existence of cysts with irregularly thickened walls. (**A**,**B**) A 39-year-old female with S-LAM. She had a left pneumothorax and the diagnosis was made histopathologically. Some of the cysts had irregularly thickened walls showing a “dot-like appearance”. (**C**) A 39-year-old female with S-LAM. Lung parenchymal radiolucency around the cysts with irregularly thickened walls in the left lung decreased compared to the right lung, suggesting lymphatic congestion in the left lung. The diagnosis of LAM was made histopathologically.

Two patients with S-LAM showed cysts with irregularly thickened walls (Fig. 2). One patient had a small number of cysts with irregularly thickened walls mixed with many cysts that had evenly thickened or thin walls (Fig. 2A,B). In the other patient (Fig. 2C), cysts with irregularly thickened walls were found only in the area with slightly increased parenchymal opacity.

#### Multiple thin-walled cysts, mostly large or irregularly shaped

**Figure 3:**
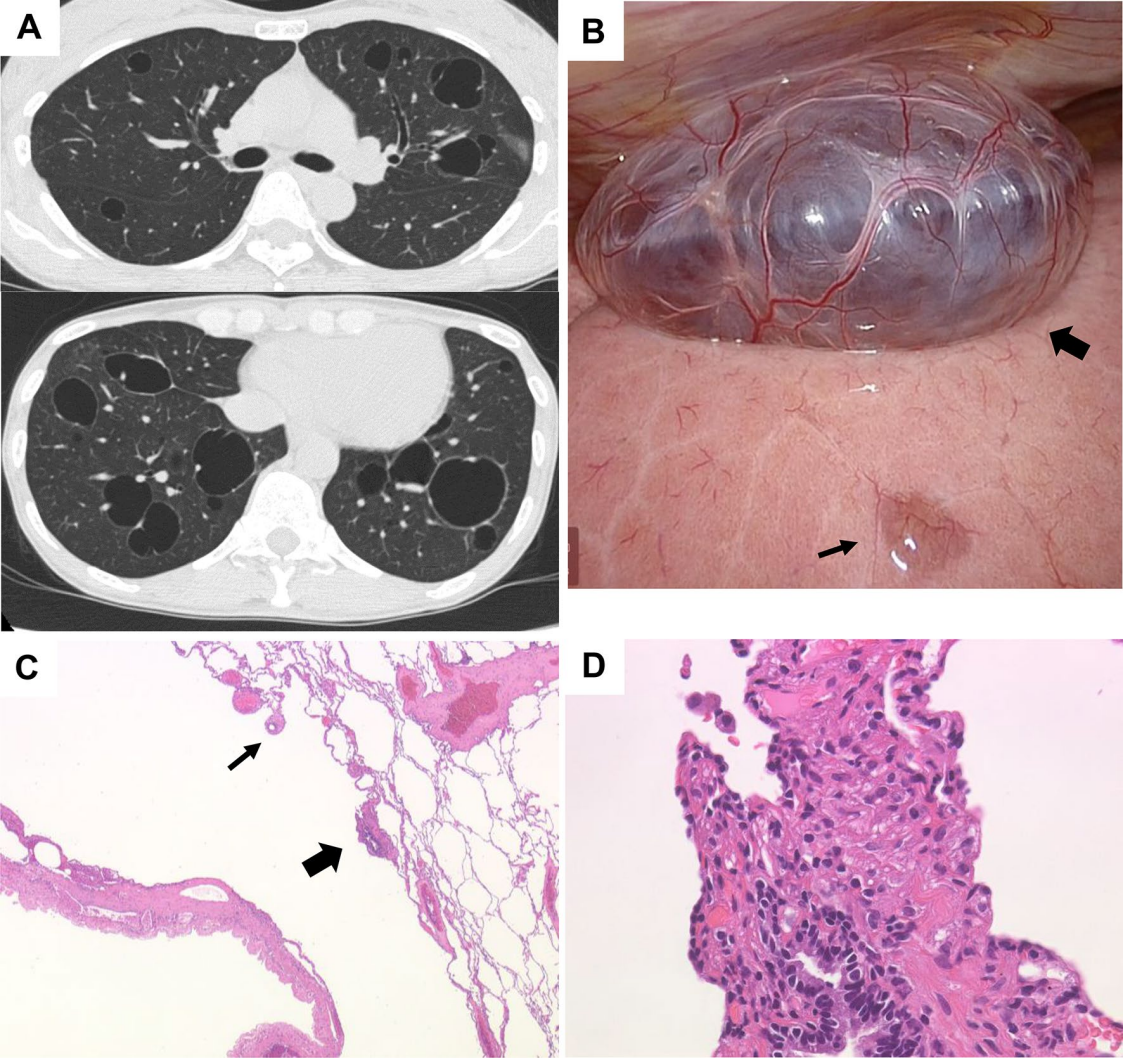
Representative HRCT images with multiple thin-walled cysts, mostly large (> 2 cm). A 44-year-old female with S-LAM. She had a history of right pneumothorax at the age of 42. Birt-Hogg-Dubé (BHD) syndrome was initially suspected from the HRCT appearance of multiple large cysts (**A**), but the *FLCN* genetic test was negative. Right pneumothorax recurrence at age 44 was treated with VATS. Thoracoscopy revealed several large transparent bullae with small blood vessels on the surface that are suggestive of BHD syndrome (**B**: thick arrow) along with tiny transparent bullae that are typical of LAM (**B**: thin arrow). Histopathological examination of the excised lung specimen revealed cysts whose walls included small scattered LAM nodules as well as bronchioles constricted by proliferating LAM cells (**C**: [arrows]; hematoxylin–eosin [HE] stain, original magnification × 25). LAM cells with short spindles or small epithelioid shapes, and pale to clear cytoplasm were roughly arrogated around bronchioles (**D**: a magnified view of area indicated by the thick arrow in **C**; HE stain, original magnification × 200). Immunohistochemical examination confirmed that LAM cells were positive for melanoma-related antigen gp100 (detected by the HMB45 monoclonal antibody), α-smooth muscle actin (α-SMA), and estrogen and progesterone receptors (data not shown).

Two patients with S-LAM showed multiple thin-walled cysts, but they were mostly large, and thus the total number of cysts in the lungs was small (Fig. 3). In this context, the differentiation from Birt–Hogg–Dubé syndrome is likely needed. We speculate that this represents check-valve mechanisms due to the bronchioles constricted by proliferating LAM cells (Fig. 3C,D), as well as comorbidity of undertreated bronchial asthma. The other patient also had an allergic predisposition, i.e., allergic rhinitis and a history of allergy to intravenous infusion of contrast material, although she had never been diagnosed as having bronchial asthma. We found only 1 S-LAM patient whose multiple thin-walled cysts were irregularly shaped. Those HRCT images have already been presented elsewhere^7^.

### Findings in addition to cysts

#### Lymphatic congestion

**Figure 4:**
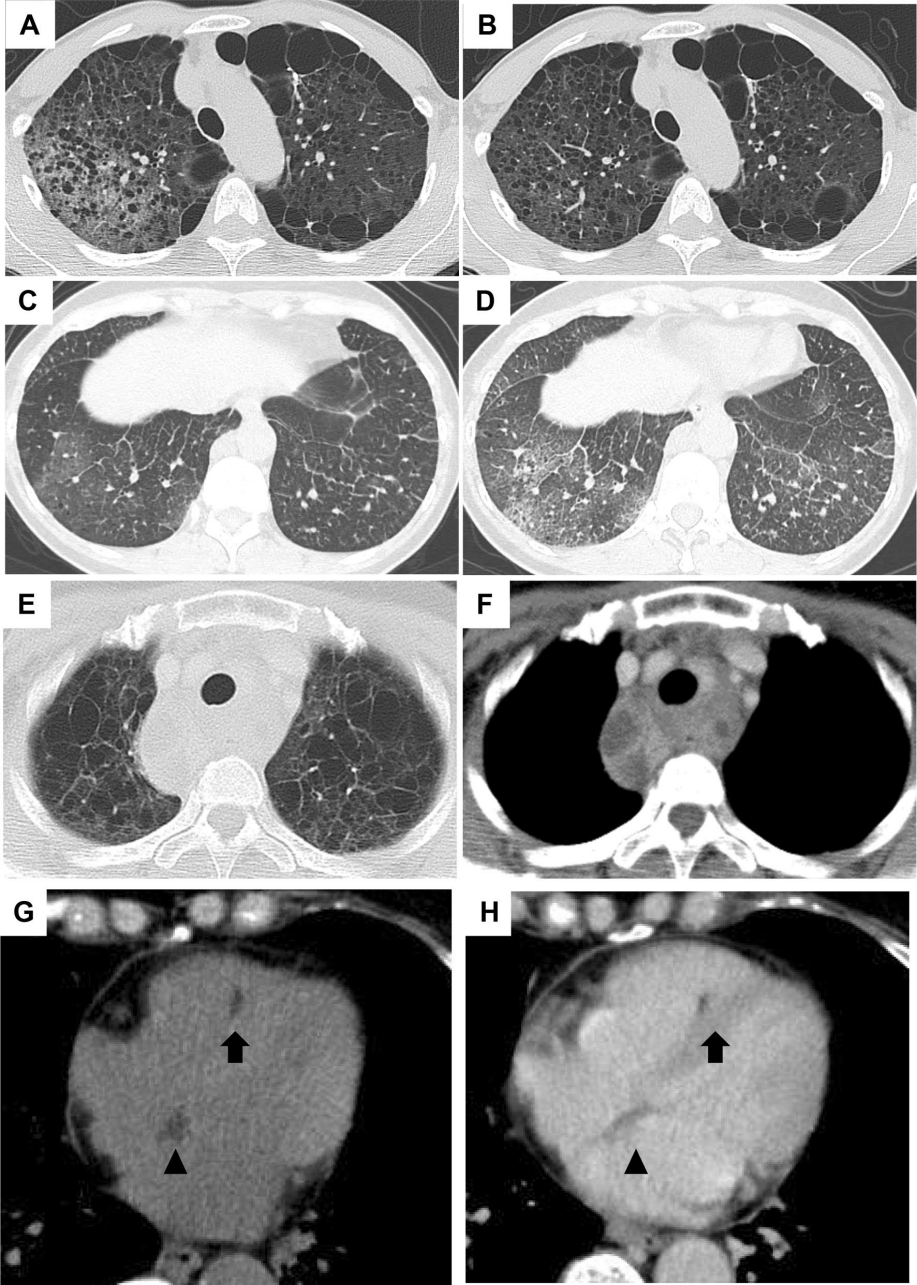
Representative HRCT images with ground-glass attenuation suggestive of lymphatic congestion and MFF. (**A**, **B**): A 53-year-old female with S-LAM. She was diagnosed as having LAM histopathologically at the age of 45 when she had a right pneumothorax. Subsequently, ground- glass attenuation was noted in the right upper lobe (**A**) which ameliorated spontaneously 3 months later (**B**). (**C**,**D**) A 48-year-old female with S-LAM. HRCT revealed a small number of cysts in lung parenchyma in which focal ground-glass attenuation and the thickening of interlobar septa were noted (**C**). These findings were suggestive of lymphatic congestion which exacerbated 3 months later (**D**). Lung biopsy confirmed the diagnosis of LAM. (**E**,**F**) Representative images of lymphatic congestion in the mediastinum. This patient was a 56-year-old female with S-LAM whose diagnosis was established histopathologically by biopsy of retroperitoneal tumors. HRCT images showed the pathognomonic cystic appearance with some large cysts due to advanced LAM disease. Note the mediastinal widening, especially of the area dorsal to the trachea (**E**). The image obtained with intravenous contrast material delineated low attenuated areas surrounded by soft tissue attenuation, suggesting the cystic dilatation of mediastinal lymphatics and/or cystic enlargement of the mediastinal lymph nodes (**F**). She had initiated a fat-restricted diet as sirolimus had not yet been approved. However, these mediastinal lesions had successfully shrunk while being on the fat-restricted diet. (**G**,**H**) A 63-year-old female with S-LAM. HRCT images showed MFF as focal low-attenuation areas in both the interatrial (arrowheads) and interventricular (arrows) septums, on both unenhanced (**G**) and enhanced (**H**) images.

Lymphatic congestion was found in 20 (7.4%) of the patients with S-LAM and 4 (10.3%) of the patients with TSC-LAM (Table 1). Lymphatic congestion was distributed in lobar, mediastinal, or limited areas. Representative HRCT images of lymphatic congestion in a limited area are shown in Fig. 4A–D. We found lymphatic congestion along the axial lymphatics in 2 patients with S-LAM. Figure 4E,F show a representative image of cystic dilatation of the mediastinal lymphatics and/or cystic enlargement of the mediastinal lymph nodes.

#### Diffuse noncalcified nodules

**Figure 5:**
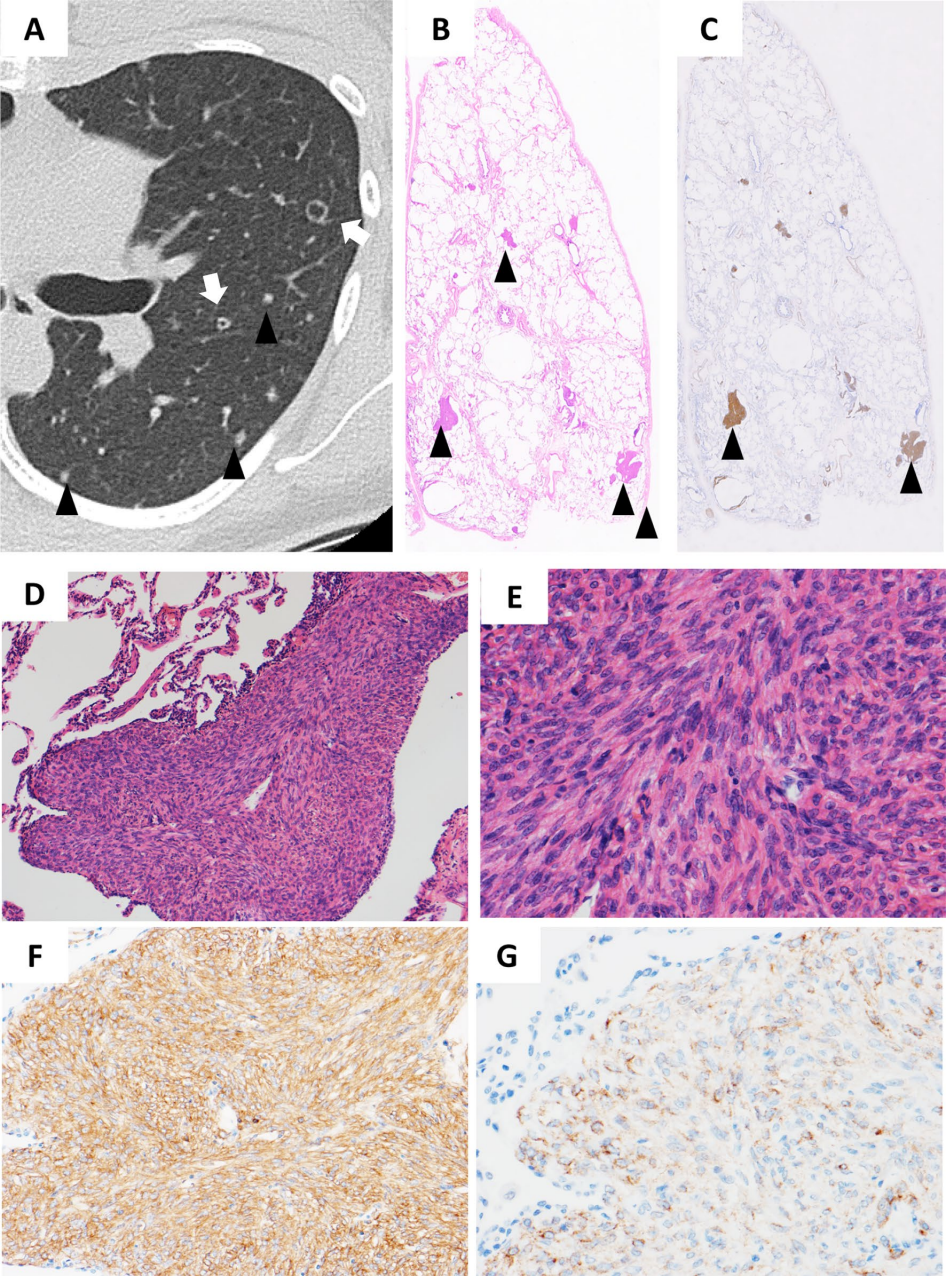
Representative HRCT images with diffuse nodules and small cavitary lesions. This patient was a 33-year-old female with S-LAM. HRCT showed diffuse nodules (arrowheads) and small cavitary lesions (white arrow) (**A**). Histopathological examination of the lung specimen obtained by VATS demonstrated that these nodules and cavitary lesions were composed of LAM lesions. A loupe view of the resected specimen showed the existence of multiple areas with nodular proliferation of LAM cells in the lung parenchyma (HE stain; arrowheads); each nodule was approximately 1.5 mm in size (**B**). Note that nodular lesions showed positive immunostaining for α-SMA (arrowheads) (**C**). The nodules were composed of uniformly proliferating spindle-shaped LAM cells and lined by alveolar epithelium (HE stain, original magnification × 90) (**D**). The magnified view showed LAM cells with eosinophilic cytoplasm and nuclei devoid of pleomorphism or mitotic activity (HE stain, original magnification × 300) (**E**). LAM cells showed cytoplasmic positive immunostaining for α-SMA (**F**: original magnification × 195) and HMB45 (**G**: original magnification × 300).

Diffuse noncalcified nodules were found in 6 (2.2%) of the S-LAM patients; four patients had diffuse nodules with small cavitary lesions, and three patients had diffuse nodules with diffuse ground-glass attenuation (one patient had both findings). No diffuse nodules were found in patients with TSC-LAM. The representative images and histopathological findings from a patient who had diffuse nodules with small cavitary lesions are shown in Fig. 5. This patient was a 33-year old female with S-LAM and histopathological examination of the lung specimen resected by video-assisted thoracoscopic surgery (VATS) confirmed that these HRCT findings were derived from LAM lesions; nodules were mainly composed of proliferating LAM cells with a paucity of LAM-associated lymphatic vessels.

**Figure 6:**
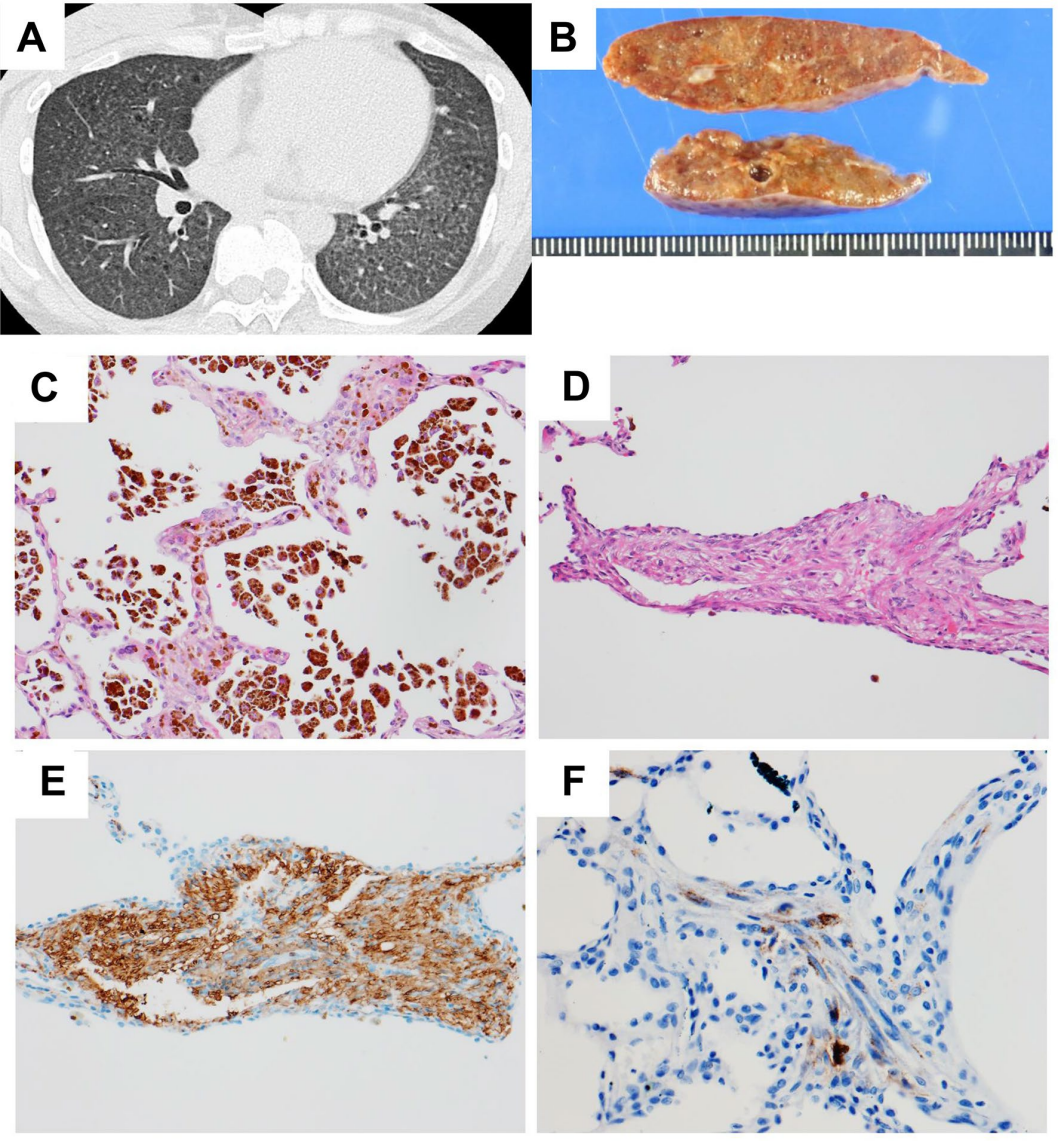
Representative HRCT images with diffuse nodules and ground-glass attenuation. This patient was a 33-year-old female with S-LAM. HRCT showed diffuse nodules with diffuse ground-glass attenuation areas (**A**). She underwent lung biopsy by VATS to establish the diagnosis. Macroscopic view of the resected specimen showed brown-colored lung tissue with a few cystic lesions (**B**). Histopathological examination demonstrated numerous hemosiderin-laden macrophages in alveolar spaces and mild thickening of the alveolar walls with hemosiderin deposition in the interstitium (**C**: HE stain, original magnification × 192). Bundles of proliferating LAM cells with slit-like spaces were also found in the lung parenchyma (**D**: HE stain, original magnification × 162). LAM cells showed cytoplasmic positive immunostaining for α-SMA (**E**: original magnification × 195) and HMB45 (**F**: original magnification × 300).

One of 3 S-LAM patients whose HRCT demonstrated diffuse nodules and ground-glass attenuation is presented in Fig. 6. This patient was another 33-year old female and histopathological examination of the lung specimen resected by VATS confirmed the diagnosis of LAM; small nodules composed of proliferating LAM cells, chronic intra-alveolar hemorrhage resulting in the accumulation of numerous hemosiderin-laden macrophages, and thickening of the alveolar wall and interstitial hemosiderin deposition were demonstrated. We speculate that the HRCT appearance, small nodules with increased parenchymal attenuation, were from the combination of these three histopathological components, with varying degrees of contribution from each.

#### TSC-related findings: rounded ground-glass opacities suggestive of multifocal micronodular pneumocyte hyperplasia (MMPH), myocardial fatty foci (MFF), and bone nodules

MMPH, MFF, and bone nodules were identified in 14 (35.9%), 18 (46.1%), and 32 (82.1%) of the TSC-LAM patients, respectively, and in 1 (0.4%), 8 (2.9%), and 28 (10.3%) of the S-LAM patients. Although the ranking of prevalence was the same in both TSC-LAM and S-LAM patients, the frequency of each finding was significantly lower in S-LAM than in TSC-LAM, *P* < 0.001 (Table 1). There were also significantly fewer MMF and bone nodules in S-LAM patients than in TSC-LAM patients (Table 2).

**Table 2:** Myocardial fatty foci and bone nodules identified on chest HRCT images.

		n	Mean	Median	Range	*P* value*
MFF	TSC-LAM	18	2.17	2	1–6	
	S-LAM	8	1.25	1	1–3	*P* < 0.05
Bone nodules	TSC-LAM	32	33.5	26.5	9–92	
	S-LAM	28	4.68	2	1–34	*P* < 0.01

*Difference between S-LAM and TSC-LAM patients (Mann–Whitney U test).

*MMF* myocardial fatty foci, *S-LAM* sporadic LAM, *TSC-LAM* tuberous sclerosis complex-associated LAM.

A representative S-LAM patient with MFF, but neither MMPH nor sclerotic bone nodules is shown in Fig. 4G,H. She had a spontaneous pneumothorax at the age of 41 and had undergone VATS for the treatment of pneumothorax. Pathological examination failed to discover LAM as the underlying disease which caused pneumothorax at that time. However, we re-evaluated the lung specimen archive when she was referred to our hospital due to a pneumothorax recurrence at the age of 63, and confirmed that she had LAM. Her HRCT revealed the typical cystic appearance with a right pneumothorax (not shown). In addition, MFF as a focal low attenuation area in the interatrial septum was observed on both unenhanced (Fig. 4G) and enhanced (Fig. 4H) images.

## Discussion

In the current diagnostic algorithm recommended by the ATS/JRC Clinical Practice Guideline^3^, “HRCT chest with features characteristic of LAM” follows as a second step after the “clinical suspicion of LAM.” Among various cystic lung diseases, LAM appears to be the most correctly diagnosed by expert radiologists and pulmonologists. Two previous studies reported accuracy rates of 72–79% in diagnosing LAM by 2 radiologists based on imaging characteristics alone^8,9^. In another study, Gupta et al. reported that the accuracy rates in diagnosing LAM based on HRCT were 91% by expert radiologists and 86% by expert pulmonologists^4^. However, it remains unclear which HRCT findings of the chest are uncommon and thus prevent pulmonologists and radiologists from considering LAM.

In this study, we found that approximately 80% of LAM patients, whether the TSC-associated or sporadic form, showed a typical or pathognomonic cystic appearance on HRCT images at their presentation or initial visit to our hospital. Interestingly, this figure seems to roughly correspond to the reported diagnostic accuracy rates^4,8,9^. The remaining patients, however, showed diverse additional findings combined with the common cystic appearance: a mixture of large cysts > 2 cm in diameter or a small number of cysts with irregularly thickened walls. Furthermore, cystic appearance was uncommon in 3 patients with S-LAM whose HRCT images were primarily composed of a countable number of large (n = 2) or irregularly shaped (n = 1) cysts. We also identified some LAM patients who presented with subtle cystic appearance but displayed diffuse noncalcified small nodules (with or without ground-glass attenuation or lymphatic congestion) that were more prominent. We believe that both clinicians and diagnostic radiologists need to realize that these additional or uncommon radiologic findings may co-exist or predominate in some LAM patients.

Cystic appearance could be modified by the occurrence of pneumothorax as well as its treatment before the clinical suspicion of LAM. Steagall et al. reported that LAM patients with a history of pneumothorax were more likely to have larger cysts on HRCT than patients who had no pneumothoraces^10^. Consistent with their study, our patients with the common cystic appearance and co-existing large cysts > 2 cm had a significantly larger proportion of pneumothorax before or at the diagnosis of LAM compared to all study participants. In addition, this subgroup tended to have a higher proportion of patients ≥ 50 years of age at presentation than all studied patients combined. This may reflect that the rate of progression of cystic destruction is slow enough so that cysts developed earlier can grow into larger cysts (see Fig. 2C,D). This notion can be supported by the recent study that found postmenopausal status to be independently associated with a lower risk of disease progression^11^.

It has been previously reported that ground-glass attenuation is often demonstrated on HRCT images of LAM patients and is considered to represent intra-alveolar hemorrhage or lymphatic congestion^7,12–15^. It would be easier to consider these radiologic findings as intra-alveolar hemorrhage or lymphatic congestion when found after the diagnosis of LAM had been established (Fig. 4A,B). However, it is not always the case (Fig. 4C,D). In the latter situation, we likely need to take a patient’s symptoms and other findings into consideration to diagnose LAM. Co-existence of thickening of alveolar septa with ground-glass attenuation, temporal fluctuation of the extent and severity of ground-glass attenuation, regression by a fat-restricted diet, and/or the occurrence of chyloptysis are likely to prompt consideration of pulmonary lymphatic congestion, whereas symptoms such as cough with hemosputa and/or anemia will lead to consideration of intra-alveolar hemorrhage^15^.

To the best of our knowledge, this retrospective study is the first to demonstrate 3 different types of uncommon HRCT appearance in LAM patients: (1) exclusively large cysts (Fig. 3); (2) multiple small nodules, some of which may appear as small cavitary lesions (Fig. 5); and (3) multiple small nodules with ground-glass attenuation in the underlying lung parenchyma (Fig. 6). In all these uncommon appearances, characteristic cystic appearance was very obscure because of the low number of thin-walled oval-shaped small cysts and/or increased parenchymal attenuation. Nodular opacities were smaller and in higher density than those suggestive of MMPH that are commonly seen in patients with TSC-LAM. All 8 patients (2.6% of all study participants) who were categorized into these 3 types of HRCT appearance had S-LAM, and were diagnosed histopathologically due to the paucity of confidence for a clinical diagnosis of LAM. The clinical suspicion of LAM based on conditions such as a young female presenting with a pneumothorax, dyspnea on exertion, or hemosputa, combined with the co-existence of a tiny number of thin-walled, oval-shaped cysts may become a driving force to propose lung biopsy for establishing the diagnosis of LAM.

S-LAM has been considered a *forme fruste* (not fully developed disease) of TSC^23^. Although MMPH, MFF, and bone nodules are currently not listed as clinical criteria for the diagnosis of TSC^5^, they have been reported to be commonly found in TSC-LAM^16–22^. Interestingly, we detected these 3 findings in S-LAM with significantly lower frequency than TSC-LAM, but with the same order of prevalence as TSC-LAM. This may further support the notion that S-LAM is a *forme fruste*, or a disease of *TSC2* mosaicism in terms of genetics. Recent genetic studies consistently demonstrated somatic *TSC1* or *TSC2* gene mutations in sporadic cases of TSC-related lesions including LAM^24,25^, renal angiomyolipoma^26^, and even focal cortical dysplasia^27^; sporadic forms of TSC-related lesions have now been considered to occur as a mosaicism of *TSC1* or *TSC2* mutations^28^.

There are several limitations in our study that could be addressed. This study is both retrospective and cross-sectional. We evaluated the HRCT images at the initial visit to our hospital for the diagnosis of multiple cystic lung diseases while suspecting LAM and conducted subsequent follow-up and treatment if necessary. Accordingly, it might inherently suffer from selection bias. However, considering the rarity of LAM, our study results still warrant attention. Second, HRCT findings might have been modified by preceding interventions, particularly in LAM patients who had developed pneumothorax. Third, the diagnosis of TSC was established according to the international consensus guideline^5^. However, we did not perform brain magnetic resonance imaging (MRI) in S-LAM patients if they had no suggestive signs or symptoms. Therefore, not all S-LAM patients who displayed MMPH, MFF, or bone sclerotic nodules were evaluated by brain MRI. However, at least one third of the S-LAM patients were confirmed to have no abnormalities by MRI.

## Conclusions

This retrospective study identified various radiologic findings that make HRCT cystic appearance uncommon of LAM. They may occur in as many as 20% of patients who are suspected to have LAM and it is important to consider them when using the proposed diagnostic algorithm for LAM.

## Supplementary Information

Supplementary Information.

## Data Availability

All data generated and/or analyzed in the current study are included in this paper and its supplementary files.

## Abbreviations

α-SMA: α-Smooth muscle actin
ATS: The American Thoracic Society
BHD: Birt–Hogg–Dubé
HE: Hematoxylin–eosin
HRCT: High resolution computed tomography
JRS: Japanese Respiratory Society
LAM: Lymphangioleiomyomatosis
MMF: Myocardial fatty foci
MMPH: Multifocal micronodular pneumocyte hyperplasia
PTX: Pneumothorax
SD: Standard deviation
S-LAM: Sporadic LAM
TSC: Tuberous sclerosis complex
TSC-LAM: Tuberous sclerosis complex-associated LAM
VATS: Video-assisted thoracoscopic surgery
VEGF-D: Vascular endothelial growth factor-D
y: Years of age
