# Supplementary material for: Uncommon radiologic computed tomography appearances of the chest in patients with lymphangioleiomyomatosis
Source: Sci Rep. 2021 Mar 30;11:7170. doi: 10.1038/s41598-021-85999-5 (PMC8010110; doi:10.1038/s41598-021-85999-5)
Supplement: Supplementary file 1 — Supplementary Information. [file 41598_2021_85999_MOESM1_ESM.pdf]

Supplementary Information

Uncommon radiologic computed tomography appearances of the chest in patients with lymphangioleiomyomatosis

Yasuhito Sekimoto [1,4], Kazuhiro Suzuki [2,4], Makiko Okura [1,4], Takuo Hayashi [3,4], Hiroki Ebana [4,5], Toshio Kumasaka [4,6], Keiko Mitani [1,4], Koichi Nishino [1,4], Shouichi Okamoto [1,4], Etsuko Kobayashi [1,4], Kazuhisa Takahashi [1] and Kuniaki Seyama [1,4]

Divisions of Respiratory Medicine [1], Radiology [2], and Human Pathology [3], Juntendo University Faculty of Medicine and Graduate School of Medicine; 3-1-3 Hongo, Bunkyo-ku, Tokyo 113-8431, Japan

The Study Group for Pneumothorax and Cystic Lung Diseases[4]; 4-8-1 Seta, Setagaya-Ku, Tokyo 158-0095, Japan

Department of Respiratory and Cardiovascular Surgery [5], Tokyo Metropolitan Bokuto Hospital; 4-23-15 Kotobashi, Sumida-ku, Tokyo 130-8575, Japan

Department of Pathology[6], Japanese Red Cross Medical Center; 4-1-22 Hiroo, Shibuyaku, Tokyo 150-8935, Japan

Corresponding author:

Yasuhito Sekimoto, M.D.

Division of Respiratory Medicine, Juntendo University Faculty of Medicine and Graduate School of Medicine; 3-1-3 Hongo, Bunkyo-ku, Tokyo 113-8431, Japan

e-mail: y-sekimo@juntendo.ac.jp

Table S1. Clinical features of LAM patients stratified by HRCT finding

HRCT Findings	n	Histologic Dx n (%)	Clinical Dx n (%)	Age at presentation			Pneumothorax n (%)
				Mean(y)	Median (Range)	≥50 n (%)	
**All patients**	**311**	**216 (69.5)**	**95 (30.5)**	**39.1**	**38 (19 - 71)**	**45 (14.5)**	**119 (38.3)**
S-LAM	272	200 (73.5)	72 (26.5)	39.2	38 (19 - 71)	39 (14.3)	112 (41.2)
TSC-LAM	39	16 (41.0)	23 (59.0)	38.3	36 (21 - 66)	6 (15.4)	7 (18.4)
**Cyst appearance**							
Common appearance alone	254	165 (65.0)	89 (35.0)	39.0	38 (22 - 67)	33 (13.0)	75 (29.5)
S-LAM	223	155 (69.5)	68 (30.5)	38.9	38 (22 - 67)	28 (12.6)	75 (33.6)
TSC-LAM	31	10 (32.2)	21 (67.7)	39.9	38 (24 - 66)	5 (16.1)	0 (0.0)
Common appearance + large cysts*	52	46 (88.5)	6 (11.5)	39.4	36 (19 - 71)	12 (23.1)[¶]	42 (80.8)[§]
S-LAM	44	40 (90.9)	4 (19.1)	40.7	38 (19 - 71)	11 (25.0)	35 (79.6)
TSC-LAM	8	6 (75.0)	2 (25.0)	32.3	32.5 (21 - 52)	1 (12.5)	7 (87.5)
Common appearance + cysts with irregularly thickened walls	2	2 (100.0)	0 (0)	NA	29, 33[†]	0	0
S-LAM	2	2 (100.0)	0 (0)	NA	29, 33[§]	0	0
TSC-LAM	0	0 (0)	0 (0)	NA	NA	0	0

Multiple thin-walled cysts, mostly large	2	2 (100.0)	0 (0)	NA	38, 43†	0	2
S-LAM	2	2 (100.0)	0 (0)	NA	38, 43†	0	2
TSC-LAM	0	0 (0)	0 (0)	NA	NA	0	0
Multiple thin-walled cysts, mostly irregularly shaped	1	1 (100.0)	0 (0)	NA	27†	0	0
S-LAM	1	1 (100.0)	0 (0)	NA	27†	0	0
TSC-LAM	0	0 (0)	0 (0)	NA	NA	0	0
**Findings in addition to cysts**							
Lymphatic congestion	24	21 (87.5)	3 (12.5)	40.5	38.5 (27 - 58)	4 (16.7)	3 (12.5)
S-LAM	20	18 (90.0)	2 (10.0)	40.3	38.5 (30 - 58)	3 (15.0)	3 (15.0)
TSC-LAM	4	3 (75.0)	1 (25.0)	41.5	41.5 (27 - 56)	1 (25.0)	0
Diffuse noncalcified nodules	6	6 (100.0)	0 (0)	38.8	33.5 (32 - 51)	2 (33.3)	3 (50.0)
S-LAM	6	6 (100.0)	0 (0)	38.8	33.5 (32 - 51)	2 (33.3)	3 (50.0)
TSC-LAM	0	0 (0)	0 (0)	NA	NA	0	0
TSC-related findings							
Rounded ground-glass opacities suggestive of MMPH	15	6 (40.0)	9 (60.0)	36.9	33 (28 - 66)	1 (6.7)	2 (13.3)
S-LAM	1	1 (100.0)	0 (0)	NA	32†	0	0

TSC-LAM	14	5 (35.7)	9 (64.2)	37.5	34 (28 - 66)	1 (7.1)	2 (14.3)
Myocardial fatty foci	26	11 (42.3)	15 (57.6)	40.7	37 (21 - 66)	7 (26.9)	7 (26.9)
S-LAM	8	6 (75.0)	2 (25.0)	45.3	49 (25 - 63)	4 (50.0)	3 (37.5)
TSC-LAM	18	5 (27.8)	13 (72.2)	38.6	36.5 (21 - 66)	3 (16.7)	4 (22.2)
Bone nodules	60	29 (48.3)	31 (51.6)	39.3	37 (21 - 67)	10 (16.7)	16 (26.7)
S-LAM	28	17 (60.7)	11 (39.3)	41.6	41 (29 - 67)	6 (21.4)	10 (35.7)
TSC-LAM	32	12 (37.5)	20 (62.5)	37.2	34.5 (21 - 66)	4 (12.5)	6 (18.8)

*Large cysts are those > 2 cm

†Indicates the age(s) at presentation when only 1-2 patients had the finding.

[¶]P=0.0809, compared to all patients (Fisher's exact test).

[§]$P<0.0001$, compared to all patients (Fisher's exact test).

Indicates the age(s) at presentation when only 1-2 patients had the finding.

Abbreviations: Dx = diagnosis; NA = not applicable; LAM = lymphangioleiomyomatosis; MMPH = multifocal micronodular pneumocyte hyperplasia; S-LAM = sporadic LAM; TSC-LAM = tuberous sclerosis complex-associated LAM; y = years of age
